# Supplementary material for: Steam Explosion-Assisted Extraction of Ergosterol and Polysaccharides from Flammulina velutipes (Golden Needle Mushroom) Root Waste
Source: Foods. 2024 Jun 13;13(12):1860. doi: 10.3390/foods13121860 (PMC11203187; doi:10.3390/foods13121860)
Supplement: Supplementary file 1 [file foods-13-01860-s001.zip › foods-3022891-supplementary.pdf]

Supplementary data

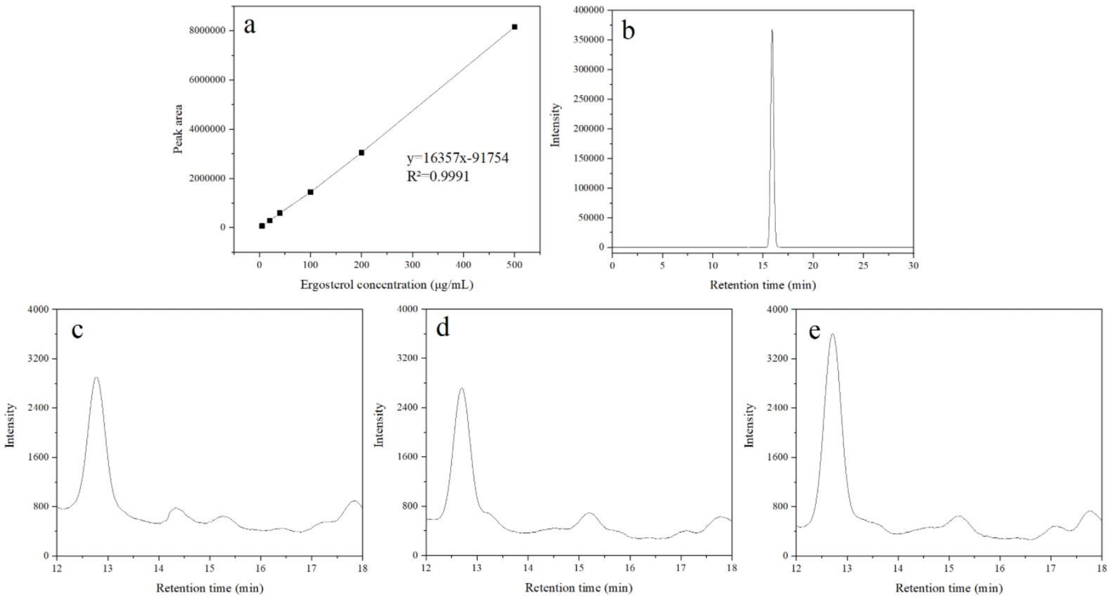

Figure S1. Standard calibration curve of ergosterol (a) and HPLC chromatography of ergosterol analysis (b: standard ergosterol; c: untreated FVR; d: steam exploded FVR at lgR=2.23; c: steam exploded FVR at lgR=3.32).

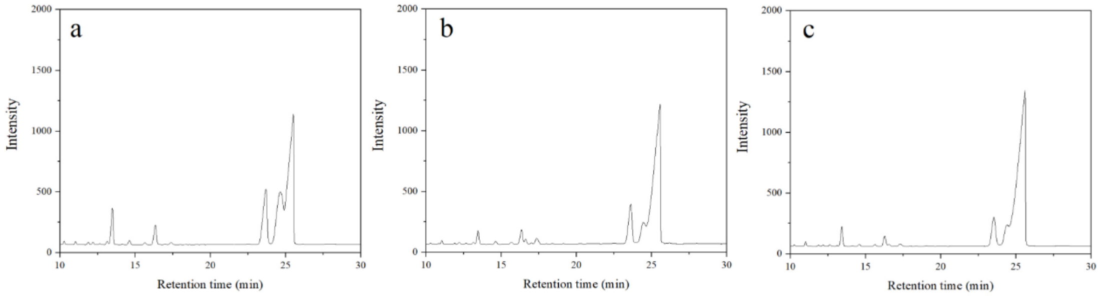

Figure S2. GC chromatography of polysaccharides from *Flammulina velutipes* roots (FVR) (a: untreated FVR; b: steam exploded FVR at lgR=3.32; c: steam exploded FVR at lgR=3.74).

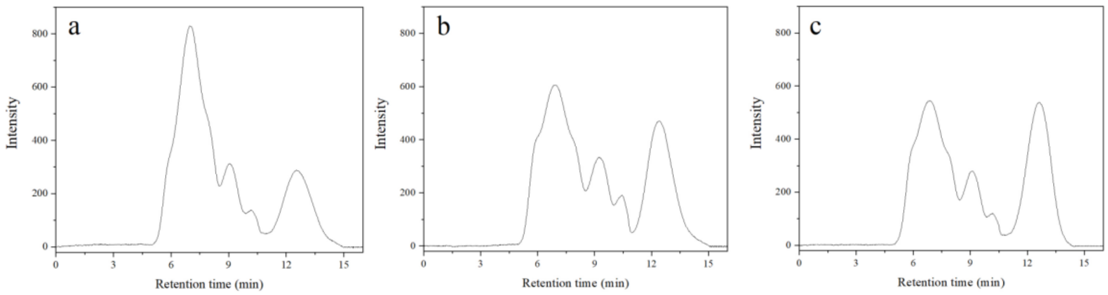

Figure S3. HPLC chromatography of molecular weight analysis (a: untreated FVR; b: steam exploded FVR at lgR=3.32; c: steam exploded FVR at lgR=3.74).
